# Significance of arterial spin labeling perfusion and susceptibility weighted imaging changes in patients with transient ischemic attack: a prospective cohort study

**DOI:** 10.1186/s12880-018-0264-6

**Published:** 2018-08-20

**Authors:** Inger Havsteen, Lasse Willer, Christian Ovesen, Janus Damm Nybing, Karen Ægidius, Jacob Marstrand, Per Meden, Sverre Rosenbaum, Marie Norsker Folke, Hanne Christensen, Anders Christensen

**Affiliations:** 1 Department of Radiology, Copenhagen University Hospital Bispebjerg, Bispebjerg Bakke 23, 2400 Copenhagen, NV Denmark; 2 Department of Neurology, Copenhagen University Hospital Bispebjerg, Bispebjerg Bakke 23, 2400 Copenhagen, NV Denmark

**Keywords:** Transient ischemic attack, Cerebral cortex, Arterial spin labeling

## Abstract

**Background:**

In a prospective cohort of patients with transient ischemic attack (TIA), we investigated usefulness and feasibility of arterial spin labeling (ASL) perfusion and susceptibility weighted imaging (SWI) alone and in combination with standard diffusion weighted (DWI) imaging in subacute diagnostic work-up. We investigated rates of ASL and SWI changes and their potential correlation to lasting infarction 8 weeks after ictus.

**Methods:**

Patients with TIA underwent 3T-MRI including DWI, ASL and SWI within 72 h of symptom onset. We defined lasting infarction as presence of 8-week MRI T2-fluid attenuated inversion recovery (FLAIR) hyperintensity or atrophy in the area of initial DWI-lesion.

**Results:**

We included 116 patients. Diffusion and perfusion together identified more patients with ischemia than either alone (59% vs. 40%, *p* < 0.0001). The presence of both diffusion and perfusion lesions had the highest rate of 8-week gliosis scars, 65% (*p* < 0.0001). In white matter, DWI-restriction was the determinant factor for scar development. However, in cortical gray matter half of lesions with perfusion deficit left a scar, while lesions without perfusion change rarely resulted in scars (56% versus 21%, *p* = 0.03). SWI lesions were rare (6%) and a subset of perfusion lesions. SWI-lesions with DWI-lesions were all located in cortical gray matter and showed high scar rate.

**Conclusions:**

ASL perfusion increased ischemia detection in patients with TIA, and was most useful in conjunction with DWI. ASL was fast, robust and useful in a subacute clinical diagnostic setting. SWI had few positive findings and did not add information.

**Trial Registration.:**

http://www.clinicaltrials.gov. Unique Identifier NCT01531946, prospectively registered February 9, 2012.

**Electronic supplementary material:**

The online version of this article (10.1186/s12880-018-0264-6) contains supplementary material, which is available to authorized users.

## Background

Diagnosing a patient with transient ischemic attack (TIA) clinically and evaluating the stroke risk hereafter [1] is a challenge even to experienced stroke neurologists [2]. The addition of noninvasive arterial spin labeling (ASL) perfusion imaging to standard TIA-MRI protocol has been shown to significantly increase the MRI detection [3] of ischemic findings. Increased reliability, [4] accessibility and automatization of post-processing have turned ASL perfusion into a clinically applicable tool [5] in stroke [6–9] and TIA [3, 10–12]. Focal ASL changes in cerebral blood flow (CBF), [13, 14] and arterial transit time, [12, 15, 16] are well described in TIA populations. [3, 10, 14] Based on ASL, lesions are estimated to occur in 35–56% [3, 17] of patients with TIA. In patients with no diffusion lesions, contrast-enhanced dynamic susceptibility contrast (DSC) perfusion imaging detects abnormalities in 16–32% [18, 19] and in 18–46% [3, 17] with both DSC and ASL. Also, perfusion changes in diffusion negative patients with clinical TIA are associated with higher rates of new diffusion weighted imaging (DWI) lesions on 3-day follow-up [20, 21]. It is conceivable that originally hypoperfused areas may progress to critical ischemia indicated by new DWI or T2-FLAIR lesions on follow-up [11, 19]. In addition, perfusion changes might also hold predictive power towards risk for de-novo future ischemic events. Consequently, the usefulness of ASL as standard add-on sequence to DWI in a subacute clinical setting without off-line post-processing is an open question.

Findings of asymmetric veins based on T2* and susceptibility weighted imaging (SWI) have also been described in stroke populations and correspond approximately to DSC hypoperfusion areas [22–25] and infarct volume at 72-h follow-up [26]. To our knowledge, SWI and findings in TIA are not described in literature. As the ischemic event in TIA is transitory and presumably less profound than in stroke, SWI may supplement ASL, reflecting venous output from ischemic tissues and indicating the depth of perfusion disturbance.

Optimizing the diagnostic yield of the various sequences included in the TIA work-up protocol is important, as since prolonged scan time decreases the quality of diagnostic evaluation due to the increased incidence of motion artefacts. Motion artefacts increase with increasing scan time, [27, 28] presence of pathology [29] and in acute settings [30].

Consequently, we aimed to investigate if standard DWI plus ASL and SWI compared to DWI alone increases the neuroradiological detection of ischemia in patients with TIA in a subacute clinical setting. In addition, we tested if ASL and SWI added to the prediction of 8-week infarction signs and recurrent cerebrovascular events after TIA.

## Methods

We studied a prospective cohort of patients with TIA admitted February 2012 – December 2014 to our comprehensive stroke center. Patients were included after own written informed consent within 72 h of symptom onset. The study was approved by the National Committee of Biomedical Research Ethics (H-1-2011-75, ClinicalTrials.gov Identifier NCT01531946).

### Clinical assessment

Senior consultant stroke neurologists clinically evaluated patients. Inclusion criteria were admission with acute focal neurological symptoms believed to be of vascular origin. Exclusion criteria were MRI contraindications, non-TIA discharge diagnosis, and severe comorbidity. We defined TIA as acutely appearing focal neurological deficit with resolution within 24 h. Resolution was defined as National Institute of Health Stroke Scale (NIHSS) 0. Patients were treated in accordance with European and national guidelines. In this study an ischemic event was defined based on either radiological signs of ischemia or clinical judgment in imaging negative patients.

Recurrent ischemic events were defined as new TIA or stroke diagnosis with clear temporal separation from the index event. We performed long-term follow-up by national electronic patient files.

### Study procedures

All patients included in this study underwent the baseline sub-acute MRI during their admission at the stroke unit. The second MRI was obtained after 8 weeks as an outpatient procedure.

### Imaging

Imaging was performed at 3T (Siemens Magnetom Verio, Siemens, Erlangen, Germany) with a 32-channel head coil (Siemens, Erlangen, Germany). The baseline imaging protocol consisted of ASL, SWI, and DWI and T2-FLAIR used in our routine TIA protocol. At 8-week follow-up MRI, we scanned only DWI and T2-FLAIR.

We used Siemens’ 3D background suppressed ep2d pulsed ASL sequence, TR 2500 ms, TE 11 ms, 192-mm FOV, FA 90, voxel size 3 × 3 × 6 mm^3^, TI1 = 700 ms, TI2 = 1800 ms, acceleration factor *R* = 2, acquisition time 4:22 min:s. Perfusion weighted images, motion corrected relative CBF and the intrinsic motion correction series were sent to PACS. The SWI protocol was TR 27 ms, TE 20 ms, 240-mm FOV, FA 15, matrix 243 × 256, 1.0 × 0.9 × 1.5 mm^3^ voxel size, 80 slices, acquisition time 5:18 min:s. ASL and SWI-postprocessing were fully automated as provided by the vendor.

The DWI was single-shot spin-echo diffusion echo-planar imaging with 220-mm FOV, 25 4-mm axial slices with 0-mm gap, b-value 0,1000 s/mm^2^ along 3 orthogonal axes; TR 6600 ms, TE 100 ms, acceleration factor R = 2, matrix 192 × 192. ADC maps were automatically generated as provided by the vendor.

For T2-FLAIR we used 240-mm FOV, 27 4-mm axial slices with 0-mm gap, TR 6500 ms, TE 133 ms, TI 2134 ms, acceleration factor R = 2, matrix 256 × 256.

### Image analysis

**Fig. 1:**
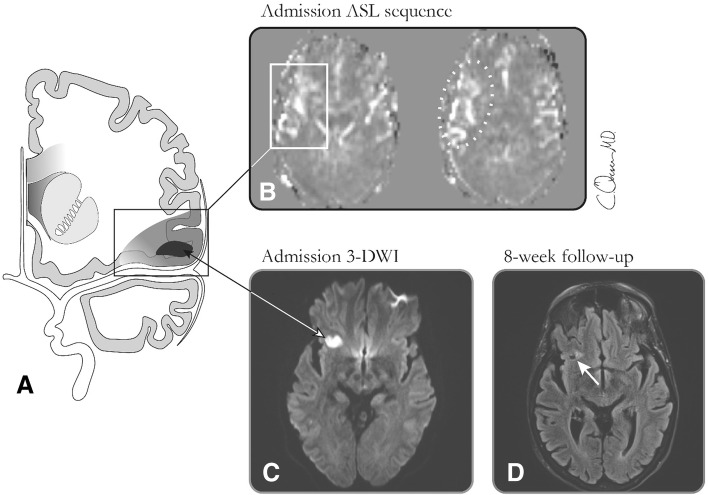
Subacute diffusion and ASL perfusion lesion and 8-week lasting infarction. Panel **a** displays a schematic illustration of infarct core with surrounding penumbra zone of perfusion restriction. Panel **b** illustrates the larger area of arterial spin labeling lesion with vivid cortical serpiginous hyperintensities (arterial transit artefact), surrounding a smaller DWI lesion (**c**). Panel **d** illustrates the permanent infarction lesion on the 8-week follow-up T2-FLAIR appearing in the same area as the DWI lesion

In this study we used only the PACS and no external software. Between sequences and MRIs performed at baseline and 8-weeks we identified lesions through their location, describing their position with two orthogonal intrathecal diameters. To provide a standardized description we developed a case report form (CRF) and literature-based image template with defined scoring categories. We validated CRF and template on 50 randomly chosen cases from the cohort with two blinded board-certified consultant neuroradiologists (AC, IH) reading first independently and then jointly establishing final consensus on the reading tools. Hereafter one reader (IH), blinded to clinical information except for the referral information, systematically assessed all baseline and 8-week imaging sequences in accordance with the predefined CRF for ASL perfusion changes, DWI-lesions, SWI asymmetric veins and missing flow voids and lesion visibility on T2-FLAIR (Fig. 1). We scored ASL, SWI and DWI images separately to avoid bias. We categorized lesion location according to vascular territory and as hemispheric left-, right, bilateral or infratentorial. For quality control we calculated intraobserver variability for two CRF readings, except for area measurements, at 3 months’ interval in a 10% sample (patients born 4th, 14th and 24th day in any month) and found substantial agreement (Cohen’s kappa = 0.80).

As motion artefacts may compromise ASL interpretation, we defined the threshold for permissible motion as motion in one plane, usually physiological motion in cranio-caudal direction, and motion presence in less than 2/3 of the whole brain series’ images. ASL image quality assessment is detailed in Additional files 1 and 2.

The arterial transit artefact (ATA) depicts areas where CBF may be normal, but where arterial transit time is increased, and is seen as vivid serpiginous high signal of labeled blood stagnated in precapillary cortical vessels [15, 16, 31]. We deemed ASL positive, when at least one of the following was present: focal low CBF, ATA or focal hyperperfusion (Fig. 1). SWI asymmetric prominent veins were defined as regions of multiple hypointense vessels [25] and dichotomized into presence and absence. We accepted newfound focal gliosis or atrophy on 8-week follow-up MRI as marker for scar tissue after neuronal death and thus critical ischemic depth [32].

We investigated lesions’ tissue localization stratifying lesions after their DWI location into white matter (WM), cortical gray matter (cGM) and deep gray matter (dGM).

After finalized clinical and radiological data collection, radiological lesion localization was compared with clinical symptoms for consistency under supervision of a senior neurological consultant (HC).

### Statistical analysis

Categorical data were analyzed using Fisher’s exact test. Differences in continuous and ordinal data were analyzed using Mann-Whitney U test, and for differences between proportions we used McNemar-test. Linear relations between variables and a dichotomous outcome were tested using bivariate logistic regression. Un-adjusted survival analysis for recurrent cerebrovascular event was conducted using Kaplan-Meier curves and log-rank test. Relation between imaging findings and recurrent cerebrovascular event was analyzed using Cox Proportional Hazard Model adjusting for other risk factors. Data were presented as frequencies, medians with interquartile range (IQR), odds ratios (OR) or hazard ratios (HR) with 95% confidence intervals (CI) as appropriate. *P*-values less than 0.05 were considered significant. Statistical analysis was performed with RStudio (Version 0.97.168) 2012 RStudio Inc., Boston, MA, USA and SPSS (version 20) statistical software (IBM Corp, Armonk, New York, USA).

## Results

### Population

**Fig. 2:**
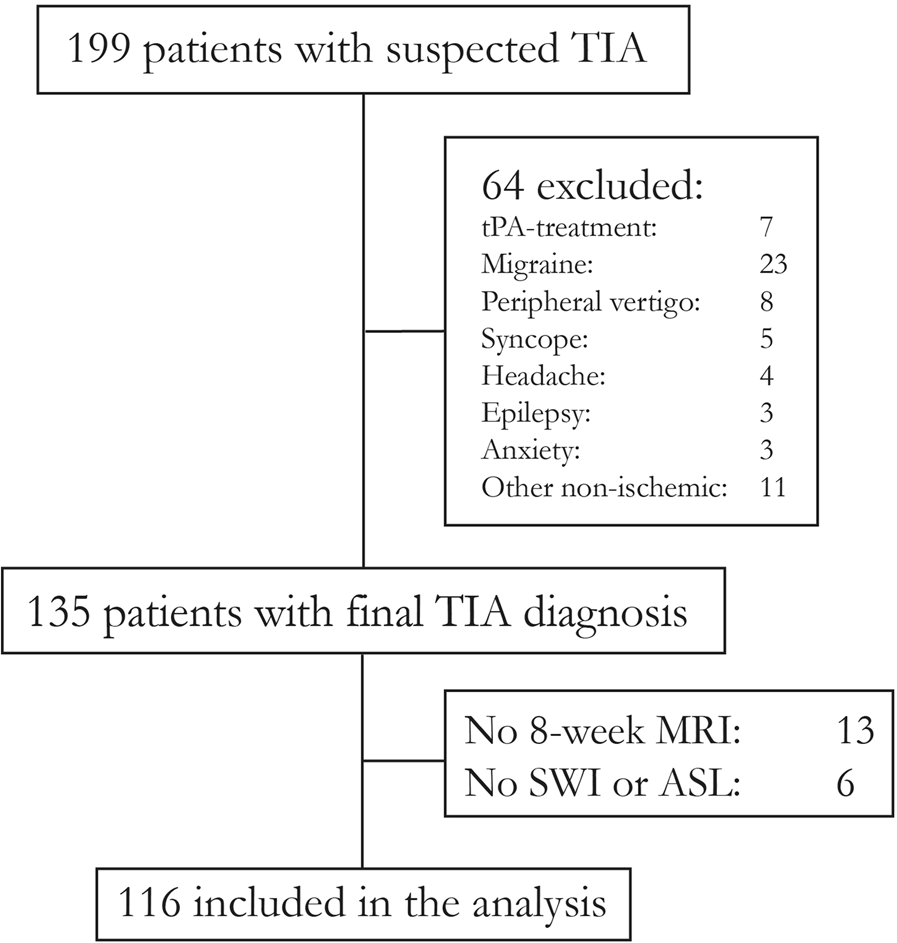
STROBE diagram of patient flow in the study

Patient flow is described in (Fig. 2). Median (IQR) age was 65 (54–71) years, 43% were female, and median (IQR) ABCD2 was 4 (3–5). Table 1 shows detailed patient characteristics. The median (IQR) time from ictus to initial MRI was 38 (24–58) hours. Scans from 4 patients had unacceptable motion artifacts, and 2 patients had no ASL done due to a technical error, these were excluded from the analysis. ASL and SWI with 8-week follow-up MRI were consequently available for 116 patients with 148 clinical or radiological events, hereof were 100 visible ischemic lesions on at least one sequence. We differentiated between patients and lesions, as patients may have multiple lesions in several vascular territories and not all foci showed perfusion changes.

**Table 1:** Patient characteristics

All patients	116
Female sex	50 (43%)
Age, median (IQR)	65 (54–71)
Medical history:	
Prior stroke	21 (17%)
Prior TIA	10 (9%)
Prior MI	9 (8%)
Atrial fibrillation	12 (10%)
Hypertension	57 (49%)
Diabetes	15 (13%)
Depression	11 (10%)
Current smoking	40 (35%)
Alcohol overuse	10 (9%)
Antiplatelet use	38 (33%)
Warfarin use	0 (0%)
Index stroke:	
ABCD2, median (IQR)	4 (3–5)
Duration of symptoms:	
< 60 min	56 (48%)
> 60 min	60 (52%)
TOAST etiology:	
Small vessels	47 (41%)
Large vessels	26 (22%)
Cardiogenic	18 (16%)
Multiple possible etiologies	25 (22%)
TTS, median (IQR)	38 (24–58)
TTF, median (IQR)	56 (55–60)
Radiological findings:	
DWI positive patients	46 (40%)
Lesions, n.	79
ASL positive patients	46 (40%)
Hypoperfusion lesions, n.	38
Hyperperfusion lesions, n.	22
ATA, n.	28
SWI positive patients	5 (4%)
SWI lesions, n.	6

Numbers are frequency (%) unless otherwise indicated. *IQR* interquartile range, *TIA* transient ischemic attack, *MI* myocardial infarction, *NA* not applicable, *TTS* time to scan, *TTF* time to follow-up, *ATA* arterial transit artefact

**Fig. 3:**
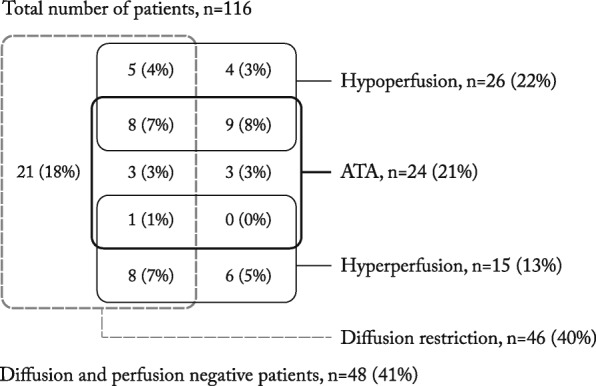
Wenn diagram of diffusion and perfusion findings in patients

Among patients 46 (40%) had at least one DWI lesion and 46 (40%) showed ASL abnormalities, 24 patients were both DWI and ASL positive (Table 1). Including both DWI and ASL as diagnostic markers of ischemia increased ischemia detection from 46 (40%) to 68 (59%) of 116 patients (*p* < 0.0001). In the cohort 148 acute clinical or radiological events were detected. Of these, 79 (53%) were DWI positive and 68 (46%) were ASL positive lesions. Diffusion and perfusion were both positive in 47 (32%) lesions and 21 lesions (14%) were ASL-positive and DWI-negative. Diffusion and ASL findings in patients are detailed in Fig. 3. Among the 68 perfusion lesions in 46 patients, ATA was present in 24 (52%, *p* < 0.0001) patients with perfusion changes and in relation to 20 (53%, *p* < 0.0001) hypoperfusion lesions. One patient with several lesions showed foci with hypoperfusion-ATA combination and foci with hyperperfusion (Fig. 3). There were no distinct lesions with discernible combinations of ATA and hyperperfusion or of hypo- and hyperperfusion.

### Characteristics of patients with 8-week infarction signs

In patients with DWI-lesions, a subsequent scarring was observed in 85% at 8 weeks. There was a significant difference between the different TOAST categories and the probability of ASL-lesion with small vessel etiology yielding the lowest probability of ASL-lesion (19%), and large vessel etiology yielding the highest probability (65%, *p* < 0.001). Adjusted for the presence of a DWI lesion, large vessel (OR 8.08, 95% CI 2.66–24.52) and multiple possible etiologies (OR 5.27, 95% CI 1.76–15.75) yielded a higher probability of ASL-lesion than either small vessel etiology (reference category) or cardiogenic etiology (OR 1.97, 95% CI 0.57–6.84). Detailed large vessel findings are shown in Additional file 1: Table S2. Adding ASL to initial DWI does not increase diagnostic accuracy of permanent infarction development (Table 2).

**Table 2:** Diagnostic accuracies of DWI and ASL findings for 8-week infarction in patients and lesions

Patients	sensitivity	specificity	PPV	NPV	accuracy
DWI+	0.98	0.91	0.85	0.99	0.93
ASL+	0.58	0.68	0.49	0.75	0.65
DWI+, ASL+	0.98	0.62	0.57	0.98	0.74
DWI+, ASL-	0.40	0.93	0.76	0.75	0.75
DWI-, ASL+	0	0.71	0	0.57	0.47
Lesions					
DWI+	0.98	0.71	0.65	0.99	0.80
ASL+	0.60	0.61	0.46	0.61	0.61
DWI+, ASL+	0.60	0.83	0.66	0.79	0.75
DWI+, ASL-	0.38	0.88	0.63	0.72	0.72
DWI-, ASL+	0	0.78	0	0.59	0.51

**Fig. 4:**
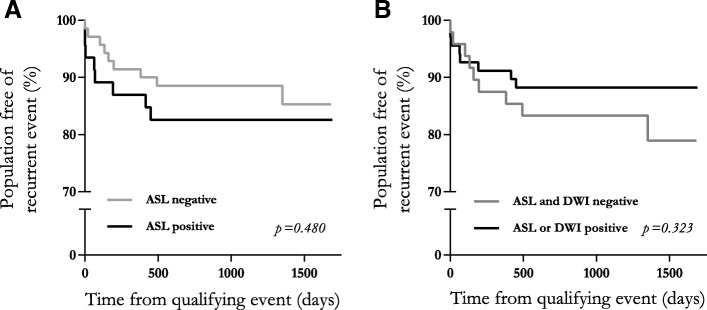
Event recurrence stratified by lesion characteristics. Two Kaplan-Meier curves showing recurrent cerebrovascular event in the TIA population stratified by MRI-sequence positivity. Panel **a** shows that patients with ASL perfusion lesions do not experience a higher risk of recurrent cerebrovascular events compared to TIA patients displaying no perfusion lesion. Panel **b**: shows that radiological evidence of acute ischemia (either restricted perfusion or diffusion) does not identify patients in higher risk of recurrent event

During the median (IQR) follow-up period of 1237 (771–1536) days, 17 patients suffered a recurrent cerebrovascular event. Patients with initial ASL lesion (Fig. 4) did not show a higher risk of recurrent cerebrovascular event (HR 1.87, 95% CI 0.70–4.97) after adjusting for presence of DWI-lesion and ABCD2.

### Characteristics of lesions with 8-week infarction signs

A total of 65% of DWI-lesions and 45% of ASL-lesions subsequently developed a scar on 8-week MRI. We found the scar-rate to be highest in lesions showing both diffusion restriction and altered perfusion (65%), however only slightly, and non-significantly higher than DWI positive and ASL negative patients (64%). The type of perfusion finding, e.g. ATA, showed no effect on the odds for persistent infarction sign development (Additional file 1: Table S3).

In WM DWI lesions had a high probability of scarring independent of the presence of perfusion abnormalities (94% vs. 92%, *p* = 0.765). In cGM DWI lesions were significantly more likely to develop subsequent infarction after 8 weeks if the lesions was ASL positive (56% vs. 21%, *p* = 0.029). The median (IQR) size of the DWI lesions located in the cGM was not significantly larger than those located in the WM (28.0 (14.0–51.0) vs. 24.5 (9.0–59.0) mm^2^; *p* = 0.463). There was a significant correlation between the size of the DWI lesion and probability of surrounding ASL lesion (OR 1.02, 95% CI 1.01–1.03 per additional mm^2^).

We had too few (*n* = 4) deep gray matter lesions to conduct a meaningful analysis.

### SWI findings

As a subset of patients with ASL abnormalities, we found 5 patients (with 6 lesions) with SWI abnormalities. In only one patient no corresponding DWI lesion was found to the ASL and SWI abnormality; this lesion did not show any scarring at the 8-week MRI. All 5 diffusion positive lesions were located in the cortical gray matter, and 4 (80%) lesions were followed by 8-week gliosis scars; this scar rate was higher than the 52% scar-rate in patients with cGM lesions with ASL abnormalities without SWI findings, though not statistically significant (*p* = 0.355). SWI abnormalities occurred both in focal hypoperfusion (2/6), ATA (2/6) and hyperperfusion (3/6).

## Discussion

This study showed that using both diffusion and ASL perfusion imaging identified significantly more patients as MRI-imaging positive for acute vascular findings than DWI alone in a subacute clinical setting without external software. DWI-lesion presence seemed to be the decisive factor for determining lasting infarct changes after TIA, especially in white matter. DWI lesions in cortical gray matter without perfusion change developed few scars, while half of lesions with perfusion change left a scar. SWI did not add to detection of ischemia in TIA, but may indicate profound perfusion disturbance.

Our study has a high frequency of lesions, most likely because of inclusion of well-defined patients and subsequent exclusion of patients with other final diagnoses than TIA. This hampers generalizability to less selected emergency room patients concerning lesion frequency, however not regarding the natural MRI history of ischemic lesions after TIA. The rates of DWI lesions are reported to vary between 25 and 50% [3, 17, 18, 33–36] and to be halved in populations with high stroke awareness and easy-access high-volume TIA clinics [37].

Other potential limitations are: Motion artifacts increase with scan time [27, 28, 38] and may compromise perfusion parameter estimates [5]. We had too few deep gray matter lesions and SWI positives for meaningful subgroup analysis. Our SWI positive lesions were a subset of perfusion positives that with concomitant DWI lesions showed high scarring rate. This may indicate that visible venous congestion serves as marker for focal ischemic depth, exploration would need more data.

We confirmed that the combination of diffusion and ASL perfusion identifies significantly more lesions than either sequence alone in TIA, [3, 14] from 40% up to nearly 60% of TIA patients in our subacute clinical setting. Roughly half of white matter lesions showed perfusion change compared to three quarters of cortical lesions: This presumably reflects the local vascularity with rich collaterals cortically and fewer in deeper tissue, relying on perforants, [39–41] and the longer and more heterogeneous transit times of white matter [42, 43] decreasing ASL’s sensitivity to focal changes. This also explains the lower probability of ASL changes in patients with small vessel etiology. Larger cortical lesions may also show signs of venous congestion, their frequency in our TIA population was scarce compared to stroke populations [25].

In our small population positive image findings did not influence the post-TIA stroke risk, nor did anyone among the 22 patients with perfusion deficits only develop infarction signs at 8-week follow-up, perhaps due to small sample size.

In our clinical setting without external post-processing software the speed and accessibility of diffusion and ASL perfusion imaging enabled us to increase ischemia detection in comparison to diffusion imaging alone. This may aid in identification of high-risk patients with TIA with imaging abnormalities. Our standard vendor pulsed ASL sequence proved robust and identified perfusion changes larger than diffusion lesions and showed perfusion change in DWI-negative patients. ATA often bordered hypoperfused areas and served as an easily identifiable pointer useful in the clinical situation, and we deemed it worth the extra scan time.

## Conclusions

Ischemia detection is improved by adding ASL to the TIA protocol, and the standard sequence proved feasible and robust in a clinical setting. SWI did not add critical information in subacute diagnostic work-up for TIA. Localization and underlying local vascularity seem to be key factors in the morphologic development of ischemia lesion depth and long-term traces.

## Additional files

Additional file 1: Supplementary methodology. Motion artefact assessment. Supplemental results. **Table S1.** Combinations of number of planes and images affected by subject motion for *n* = 116 patients. **Table S2.** Large vessel findings for patients (*n* = 116) stratified after most likely etiology after full radiological and clinical workup. **Table S3.** Risk of persistent infarction signs for DWI lesions and perfusion findings. (DOCX 20 kb)

Additional file 2: **Figure S1.** ASL interpretability and artefact description for 116 patients. Top 4 rows compare ASL PWI and relCBF images. Bottom row shows ring artefact frequency. GW = gray-white matter discrimination. (PDF 4 kb)

## Abbreviations

ASL: Arterial spin labeling
ATA: Arterial transit artefact
CBF: Cerebral blood flow
cGM: Cortical gray matter
CI: Confidence interval
CRF: Case report form
dGM: Deep gray matter
DSC: Dynamic susceptibility contrast
DWI: Diffusion weighted imaging
FLAIR: Fluid attenuated inversion recovery
HR: Hazard ratio
IQR: Interquartile range
MRI: Magnetic resonance imaging
NIHSS: National Institutes of Health Stroke Scale
OR: Odds ratio
SWI: Susceptibility weighted imaging
TIA: Transient ischemic attack
TOAST: Trial of ORG 10172 in acute stroke treatment
WM: White matter
