# Supplementary material for: Significance of arterial spin labeling perfusion and susceptibility weighted imaging changes in patients with transient ischemic attack: a prospective cohort study
Source: BMC Med Imaging. 2018 Aug 20;18:24. doi: 10.1186/s12880-018-0264-6 (PMC6102826; doi:10.1186/s12880-018-0264-6)
Supplement: Supplementary file 1 — Supplementary methodology. Motion artefact assessment. Supplemental results. Table S1. Combinations of number of planes and images affected by subject motion for n = 116 patients. Table S2. Large vessel findings for patients (n = 116) stratified after most likely etiology after full radiological and clinical workup. Table S3. Risk of persistent infarction signs for DWI lesions and perfusion findings. (DOCX 20 kb) [file 12880_2018_264_MOESM1_ESM.docx]

**Additional file**

Significance of arterial spin labeling perfusion and susceptibility weighted imaging changes in patients with transient ischemic attack

**Havsteen, et al. 2018**

Supplemental methodology:

Motion artefact assessment:

We assessed motion artefacts as a combination of scores for the number of affected planes and images in the ASL whole brain series consisting of 16 slices. The number of planes affected by motion we graded as 0=still, 1=physiologic, mainly cranio-caudad nodding-like motion while breathing, 2+= nodding and head wagging (‘maybe’) or rotation (‘no-no’). The number of images in the whole brains series affected by motion we graded by thirds, 0≤1/3 of images; 1=1/3-2/3 images; 2+≥2/3. The combination (2,2) was deemed unacceptable.

Our pulsed ASL sequence presented in the PACS both PWI and M0-corrected relCBF images. M0-correction holds assumptions on blood transit time and may potentially introduce a systematic error if the PASL inversion time does not match blood transit time. We compared degree of artefacts and gray-white matter discrimination on PWI and relCBF images.

The ASL whole brain series consisted of 16 slices. We characterized ASL motion artefacts and described the extent of the images affected for the central 12 and 4 peripheral slices. We used Kruskal-Wallis test to assess for association between motion artefact grading and presence or absence of long-term infarction signs.

Supplemental results:

In this population we found no patients without motion artefacts on ASL and the majority (91%, 106/116 patients) showed mild physiological artefacts. Suppl. Figure shows that ASL PWI and relCBF images concurred on degree of artefacts and gray-white matter discrimination and artefact presence was most pronounced in the peripheral slices of the ASL whole brain series. Ring artefact was present in 84% (97/116) of patients. We found no association between presence of persistent infarction signs and motion artefact grading severity (p=0.37, Suppl. Table 1).

Supplemental tables:

Table S1 Combinations of number of planes and images affected by subject motion for n=116 patients.

| Motion grading (planes, images) | All (n=116) | Infarct (n=40) | No infarct (n=76) |
| --- | --- | --- | --- |
| (1,1) | 24 | 6 | 18 |
| (1,2) | 82 | 32 | 50 |
| (2,1) | 10 | 2 | 8 |

Table S2 Large vessel findings for patients (n=116) stratified after most likely etiology after full radiological and clinical workup. All patients received carotid Doppler, *CTA was available for 69 patients.

| Etiology | N | Ipsilateral extracranial stenosis | Contralateral extracranial stenosis | Ipsilateral intracranial stenosis* | Contralateral intracranial stenosis* |
| --- | --- | --- | --- | --- | --- |
| Small vessel | 47 | 0 | 0 | 0/23 | 0/23 |
| Large vessel | 26 | 2 | 1 | 3/20 | 0/20 |
| Cardiac | 18 | 0 | 0 | 0/10 | 0/10 |
| Multiple possible etiologies | 25 | 2 | 5 | 6/16 | 0/16 |

Table S3 Risk of persistent infarction signs for DWI lesions and perfusion findings.

| Characteristics | # lesions | # lesions with persistent infarction signs (%) | # lesions with no persistent infarction signs (%) | p | OR (95% CI) |
| --- | --- | --- | --- | --- | --- |
| DWI only | 32 | 20 (63) | 12 (38) | 0.81 | 0.86 (0.34-2.19) |
| DWI+ hypo | 14 | 8 (57) | 6 (43) | 0.55 | 0.69 (0.18-2.71) |
| DWI+hypo+ ATA | 11 | 7 (64) | 4 (36) | 1 | 0.96 (0.22-4.91) |
| DWI+ATA | 5 | 5 (100) | 0 | 0.15 | - |
| DWI+hyper | 17 | 11 (65) | 6 (35) | 1 | 1.01 (0.29-3.80) |
| Lesion total | 79 | 51 (65) | 28 (35) | - | - |
| DWI+  any ATA | 16 | 12 (75) | 4 (25) | 0.39 | 1.83 (0.48-8.70) |
| DWI + any hypo | 25 | 15 (60) | 10 (40) | 0.62 | 0.75 (0.25-2.27) |

Supplemental figure:

Figure S1(Additional file 2). ASL interpretability and artefact description for 116 patients.

Top 4 rows compare ASL PWI and relCBF images. Bottom row shows ring artefact frequency. GW=gray-white matter discrimination.
