# Supplementary figures and images for: Significance of arterial spin labeling perfusion and susceptibility weighted imaging changes in patients with transient ischemic attack: a prospective cohort study
Source: BMC Med Imaging. 2018 Aug 20;18:24. doi: 10.1186/s12880-018-0264-6 (PMC6102826; doi:10.1186/s12880-018-0264-6)

■ No    □ Yes

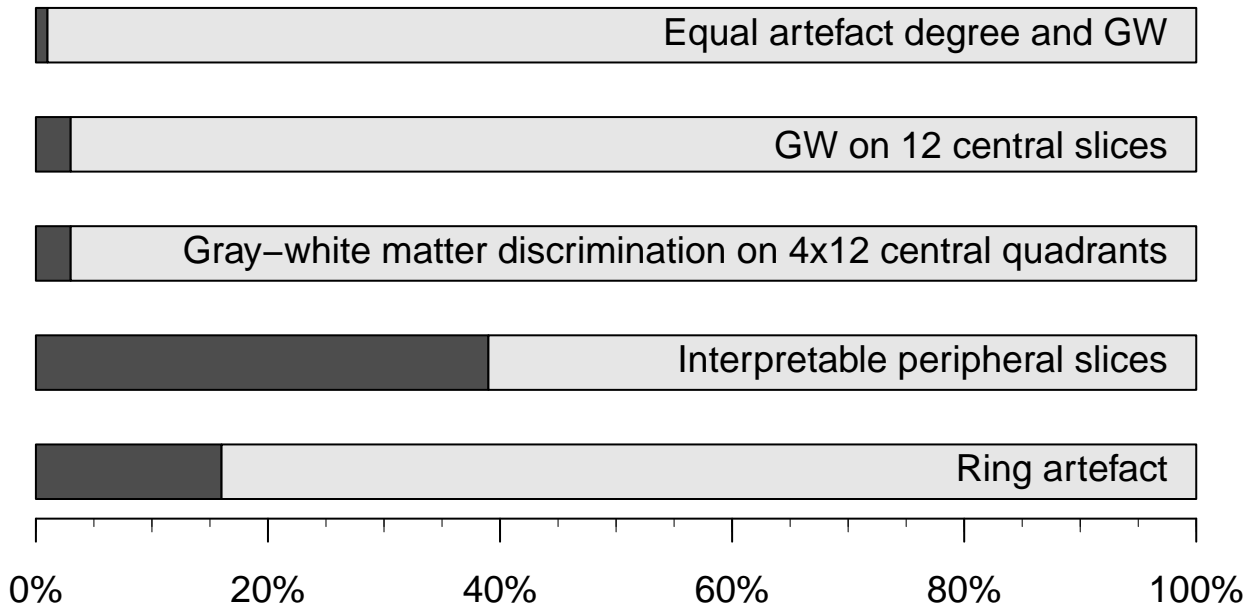

Supplement: Supplementary file 2 — Figure S1. ASL interpretability and artefact description for 116 patients. Top 4 rows compare ASL PWI and relCBF images. Bottom row shows ring artefact frequency. GW = gray-white matter discrimination. (PDF 4 kb) [file 12880_2018_264_MOESM2_ESM.pdf]
